# Analysis of strain, sex, and diet-dependent modulation of gut microbiota reveals candidate keystone organisms driving microbial diversity in response to American and ketogenic diets

**DOI:** 10.1186/s40168-023-01588-w

**Published:** 2023-10-03

**Authors:** Anna C. Salvador, M. Nazmul Huda, Danny Arends, Ahmed M. Elsaadi, C. Anthony Gacasan, Gudrun A. Brockmann, William Valdar, Brian J. Bennett, David W. Threadgill

**Affiliations:** 1 Department of Molecular and Cellular Medicine, Texas A&M Health Science Center, College Station, TX 77843 USA; 2 Department of Nutrition, Texas A&M University, College Station, TX 77843 USA; 3 Department of Nutrition, University of California Davis, Sacramento, CA 95616 USA; 4 Obesity and Metabolism Unit, Western Human Nutrition Research Center, USDA-ARS, Davis, CA 95616 USA; 5 Albrecht Daniel Thaer-Institut, 10115 Berlin, Germany; 6 Department of Applied Sciences, Northumbria University, Newcastle Upon Tyne, UK; 7 Department of Genetics, University of North Carolina at Chapel Hill, Chapel Hill, NC 27599 USA; 8 Lineberger Comprehensive Cancer Center, University of North Carolina at Chapel Hill, Chapel Hill, NC 27599 USA; 9 Department of Biochemistry & Biophysics, Texas A&M University, College Station, TX 77843 USA

**Keywords:** mouse, diet, keystone species, microbiome, ketogenic

## Abstract

**Background:**

The gut microbiota is modulated by a combination of diet, host genetics, and sex effects. The magnitude of these effects and interactions among them is important to understanding inter-individual variability in gut microbiota. In a previous study, mouse strain-specific responses to American and ketogenic diets were observed along with several QTLs for metabolic traits. In the current study, we searched for genetic variants underlying differences in the gut microbiota in response to American and ketogenic diets, which are high in fat and vary in carbohydrate composition, between C57BL/6 J (B6) and FVB/NJ (FVB) mouse strains.

**Results:**

Genetic mapping of microbial features revealed 18 loci under the QTL model (i.e., marginal effects that are not specific to diet or sex), 12 loci under the QTL by diet model, and 1 locus under the QTL by sex model. Multiple metabolic and microbial features map to the distal part of Chr 1 and Chr 16 along with eigenvectors extracted from principal coordinate analysis of measures of β-diversity. *Bilophila*, *Ruminiclostridium 9*, and *Rikenella* (Chr 1) were identified as sex- and diet-independent QTL candidate keystone organisms, and *Parabacteroides* (Chr 16) was identified as a diet-specific, candidate keystone organism in confirmatory factor analyses of traits mapping to these regions. For many microbial features, irrespective of which QTL model was used, diet or the interaction between diet and a genotype were the strongest predictors of the abundance of each microbial trait. Sex, while important to the analyses, was not as strong of a predictor for microbial abundances.

**Conclusions:**

These results demonstrate that sex, diet, and genetic background have different magnitudes of effects on inter-individual differences in gut microbiota. Therefore, Precision Nutrition through the integration of genetic variation, microbiota, and sex affecting microbiota variation will be important to predict response to diets varying in carbohydrate composition.

Video Abstract

**Supplementary Information:**

The online version contains supplementary material available at 10.1186/s40168-023-01588-w.

## Introduction

The gut microbiota has emerged as a key component underlying the application of precision nutrition and individualized dietary response. Gut microbiota utilizes nutrients passing through the gastrointestinal tract to perform biological functions, this in turn impacts host digestion, absorption, and metabolism of nutrients [1]. There is a consensus that a relationship exists between the microbes and their host. Although some studies have been performed in humans and livestock species, e.g., quails and hens [2, 3], the impact of inter-individual variability on how diet modulates gut microbiota composition remains underinvestigated [4, 5].

Previous studies from our group have demonstrated strong mouse strain-specific differences in response to American and ketogenic diets [6–9], especially between the C57BL/6 J (B6) and FVB/NJ (FVB) strains. The composition of gut microbiota is known to be influenced by both host genetics and environmental factors such as diet [9–11], which is considered one of the most potent regulators of gut microbial composition. We have recently demonstrated that B6 is particularly susceptible to altered gut microbiota relative to A/J, FVB, and NOD/ShiLtJ [9]. Furthermore, changes to bacterial abundance do not occur uniformly in response to diets varied in macro- and micronutrient composition because of differences in substrate utilization between bacterial taxa [9, 12]. To determine what the composition of the “ideal” microbiome is, it would be pertinent to disentangle the effects of host genetics and host diet from the extra layers of complexity arising from differences in substrate utilization by individual organisms and ultimately identify genes regulating interindividual differences in the composition of the gut microbiota [12, 13]. Until recently, few studies have considered the extent to which the combination of host genetics and diet modulate the abundance of specific bacterial taxa, and even fewer have considered how sex might add an additional layer of complexity to describing inter-individual variation in microbiota composition [14–18].

In this study, an intercross population (F2) was generated between B6 and FVB to investigate the strain-, sex-, and diet-dependent modulation of the gut microbiota. F2s were fed either an American or a ketogenic diet and fecal microbiota was quantified. The results provide evidence for 32 quantitative trait loci (QTL) that affect microbiota composition, but also significant diet and sex differences in the effect size of the QTL. In many cases, these were sex- and diet-independent QTL (i.e., marginal effect QTL that are not specific to diet or sex, y ~ sex + diet + sex:diet + [marker]), and in other cases, these were genotype- and diet-dependent (y ~ sex + diet + marker + [marker:diet]) or genotype- and sex-dependent QTL (y ~ sex + diet + marker + [marker:sex]), which allowed us to characterize how much host genetics, sex, and diet affect specific gut microbiota, and provided insights into factors driving microbial diversity, which has implications for advancing precision nutrition through preclinical studies.

## Methods

### Animals and diets

B6 females were crossed with FVB males to generate F1 mice and subsequently intercrossed to generate an F2 population. Both parental strains were acquired from The Jackson Laboratory prior to generating the F2 population. F2s were randomized to five mice per cage and screened for their response to American (a powdered meal composed of 35% of energy from fat, 50% from carbohydrates) and ketogenic (a paste composed of 84% of energy from fat, 0% from carbohydrates) diets during a 3-month feeding trial. Neither diet was irradiated prior to use. Detailed diet compositions are provided in Supplementary Table S1.

For the feeding trials, 3–5 week-old mice were randomly assigned to one of the two diet groups and allowed to eat ad libitum. Half of the F2 mice were placed on American diet (102 males, 122 females) and half on ketogenic diet (126 males, 119 females). All protocols in this study were approved by the Texas A&M University Institution Animal Care and Use Committee (IACUC protocol number: 2022–0273) and animals were maintained in accordance with those guidelines at 22 °C under a 12-h light cycle with up to 5 mice per cage, in compliance with the Guide for the Care and Use of Laboratory Animals published by the US National Institutes of Health. Detailed husbandry descriptions are provided in Supplementary Table S2 per ARRIVE guidelines for reporting housing and husbandry conditions. At the end of the feeding trial, mice were euthanized by carbon dioxide asphyxiation, blood was collected, and tissues and feces were harvested and immediately flash frozen in liquid nitrogen.

### Microbiota phenotypes

Stool microbiota was analyzed by 16S rRNA V4 sequencing methodology as reported previously [19]. In brief, total stool DNA was extracted using ZymoBIOMICS™ 96 MagBead DNA kit (Zymo Research, Irvine, CA) with an automated epMotion (Eppendorf, Hamburg, Germany) robotic system. About 100 mg of stool samples were placed in the ZR BashingBead™ Lysis Tube and homogenized using FastPerp24 bead beater (Millipore, Hayward, CA) at 6.5 HZ for 2 min. The lysate was centrifuged at ≥ 10,000 × g for 1 min and 200 μl supernatant from lysis tube was transferred to 96 deep-well plate (Eppendorf, Hamburg, Germany) and loaded in an epMotion 5075t robotic system. Using epMotion, 600 μl ZymoBIOMICS™ MagBinding Buffer and 25 μl of ZymoBIOMICS™ MagBinding Beads were added to each well and was mixed well. After mixing, the plate was placed on a magnetic stand and the supernatant was discarded. MagBinding Beads were washed with MagWash 1 and MagWash 2 and the DNA was eluted using 50 μl ZymoBIOMICS™ DNase/RNase free water. The DNA concentration was measured using NanoDrop One (Thermo Scientific, Petaluma, CA).

Mixed template amplicon library for the 16S variable region 4 (V4) was prepared according to the protocol from the Earth Microbiome Project (EMP_ (http://www.earthmicrobiome.org/emp-standard-protocols/) using the extracted stool total DNA and the primer sets (515F and barcoded 806R) [20]. The PCR master mix, primer, and samples were plated using the epMotion. Appropriate NTC, extraction control, and pooled fecal sample were added to each plate. The PCR master mix was prepared consisting of 37.5 µl of GoTaq Green Master Mix (Promega, Madison, WI), 3 µl of 25 mM MgCl_2_, 1.5 µl of 10 µM forward primer 515F, and 25.5 µL of nuclease-free water. Then, 1.5 µl of 10 µM barcode-specific reverse primer 806R and 6 µl of extracted stool DNA were added. PCR was performed in duplicate of 25µL under the following conditions: denaturation (1 cycle) at 94 °C for 3 min; amplification of 25 cycles at 94 °C for 45 s, 50 °C for 60 s, and 72 °C for 90 s; and a final extension step cycle at 72 °C for 10 min. Amplicon DNA was multiplexed and purified using Wizard SV Gel and PCR Clean-Up System (Promega, Madison, WI). The amplicon library was sequenced using the Illumina MiSEQ platform with 2 × 250 bp paired-end sequencing. Sequences were de-multiplexed and exact amplicon sequence variants (ASV) in the 16S rRNA gene sequence were determined using the open-source software QIIME2-DADA2 pipeline [21]. A total of 11,316,115 sequences with an average of 26,074 ± 13,697 (mean ± SD) sequences per sample were recovered after demultiplexing. Taxonomy was assigned using the SILVA 132 reference database [22] customized for 16 s V4 (515F/806R) region of sequences at the threshold of 99% pairwise identity. ASV belonging to mitochondria and chloroplast were filtered out from the ASV table. We performed a single rarefaction at a sequence depth of 4,500 sequences per sample. α-diversity (Shannon diversity index and observed species) and β-diversity (unweighted UniFrac, weighted UniFrac, Jaccard Index, and Bray Curtis) were calculated from the unfiltered ASV table. Any ASV not seen more than 5 times in at least 5% of the samples were removed for calculating differential bacteria abundance. 16S V4 Sequences are publicly available on the SRA database under the Bioproject ID “PRJNA803237.”

Microbial traits are listed by both ASV ID and maximum taxonomic information for reference in Table 1. The rank of the maximum taxonomic information is described at first mention of all microbial traits within the text and indicated with a taxonomic rank in subsequent figures and tables (i.e., D0, Kingdom; D1, Phylum; D2, Class; D3, Order; D4, Family; D5, Genus; D6, Species).

**Table 1:** Linkage analysis for microbial traits in the combined, diet-specific, and sex-specific models

Phylum	ASV ID	Maximum taxonomic information	Model	Left marker	Top marker	Right marker	QTL identifier	Chr	Start (Mb)
Actinobacteria	87f436ffd9de6eb9451032c6ab0c3a7e	*D_5__Coriobacteriaceae UCG-002*	Combined y ~ m + sex*diet	JAX00664614	backupUNC08018	UNC080237919	*Asvq1*	8	31.0
Actinobacteria	11c66263fce8e3ed0b6f024451a3e4f6	*D_5__Enterorhabdus*	Combined y ~ m + sex*diet	JAX00160641	JAX00668481	UNC080591069	*Asvq2*	8	36.1
Bacteroidetes	b638b1c18c30ded1d2df7558b49ee113	*D_4__Muribaculaceae;D_5__uncultured bacterium*	Combined y ~ m + sex*diet	UNC140463041	backupUNC14027	UNC140475528	*Asvq3*	14	74.3
Bacteroidetes	4e591c10e196ae155d20629952d85e34	*D_4__Muribaculaceae;D_5__uncultured bacterium*	Combined y ~ m + sex*diet	JAX00430230	UNC170287409	backupUNC1700	*Asvq4*	17	5.6
Bacteroidetes	2cc64f7f2dcfc098d317347e8d3323aa	*D_4__Muribaculaceae;D_5__uncultured bacterium*	Combined y ~ m + sex*diet	JAX00430230	UNC170287409	backupUNC1700	*Asvq5*	17	5.6
Bacteroidetes	7738a54ecc2a9056c502e19953a34adb	*D_5__Alistipes*	Combined y ~ m + sex*diet	backupJAX00343	UNC120435288	backupUNC1202	*Asvq6*	12	101.9
Bacteroidetes	ecfbbfd673f8a3e1c34718d968e6b069	*D_5__Rikenella*	Combined y ~ m + sex*diet	JAX00271510	UNC010409945	UNC010872195	*Asvq7*	1	151.9
Bacteroidetes	4cd0cd5a04c3abd39385cc354976ff4d	*D_5__Rikenellaceae RC9 gut group*	Combined y ~ m + sex*diet	UNC110054975	UNC110464797	JAX00311806	*Asvq8*	11	42.9
Firmicutes	8ab9ece30db28dd09f0e76f426a709c9	*D_5__Streptococcus*	Combined y ~ m + sex*diet	UNC080433052	UNC080671076	backupUNC0803	*Asvq9*	8	108.8
Firmicutes	8ab9ece30db28dd09f0e76f426a709c9	*D_5__Streptococcus*	Combined y ~ m + sex*diet	UNC080237919	JAX00671644	UNC080301746	*Asvq10*	8	58.6
Firmicutes	be6ee4f4c44adbe6834438d109b75853	*D_5__GCA-900066575*	Combined y ~ m + sex*diet	JAX00172457	JAX00173057	UNC090299874	*Asvq11*	9	68.4
Firmicutes	403f6d9b8d155413c40a76568324cf95	*D_5__Lachnoclostridium*	Combined y ~ m + sex*diet	JAX00005015	UNC010587748	backupUNC0102	*Asvq12*	1	70.1
Firmicutes	a122e3f0437bb8880bab1660d8e0c289	*D_5__Lachnoclostridium*	Combined y ~ m + sex*diet	JAX00042454	UNC130039434	backupUNC1305	*Asvq13*	13	21.2
Firmicutes	56f81e7c72e8c90daafad2ffad595810	*D_5__Romboutsia*	Combined y ~ m + sex*diet	UNC050381284	UNC050154313	backupUNC0506	*Asvq14*	5	66.1
Firmicutes	56f81e7c72e8c90daafad2ffad595810	*D_5__Romboutsia*	Combined y ~ m + sex*diet	UNC130005651	backupJAX00351	4UNC130039434	*Asvq15*	13	5.4
Firmicutes	b89e2f23a606787577644904f2b6aed3	*D_5__Ruminiclostridium 9*	Combined y ~ m + sex*diet	UNC_rs3360795	8backupUNC01062	backupJAX00013	*Asvq16*	1	138.0
Proteobacteria	18f60eae2c3a26bab8efa0f9823e31b4	*D_5__Bilophila*	Combined y ~ m + sex*diet	UNC010463165	UNC010409945	UNC010872195	*Asvq17*	1	144.5
Proteobacteria	18f60eae2c3a26bab8efa0f9823e31b4	*D_5__Bilophila*	Combined y ~ m + sex*diet	backupUNC0900	6JAX00173057	UNC090299874	*Asva18*	9	42.3
Phylum	ASV barcode	Maximum taxonomic information	Model	Left marker	Top marker	Right marker	QTL identifier	Chr	Start (Mb)
Bacteroidetes	4e591c10e196ae155d20629952d85e34	*D_4__Muribaculaceae;D_5__uncultured bacterium*	Diet specific y ~ sex + diet*m	JAX00351389	JAX00042454	UNC130039434	*Asvq19*	13	18.2
Bacteroidetes	2cc64f7f2dcfc098d317347e8d3323aa	*D_4__Muribaculaceae;D_5__uncultured bacterium*	Diet specific y ~ sex + diet*m	UNC130013342	backupUNC13002	UNC130039434	*Asvq20*	13	12.5
Bacteroidetes	34a3b7dc9cbbb1e182c7f1085f289288	*D_4__Muribaculaceae;D_5__uncultured bacterium*	Diet specific y ~ sex + diet*m	UNC140463041	UNC140471593	UNC140475528	*Asvq21*	14	74.3
Bacteroidetes	b638b1c18c30ded1d2df7558b49ee113	*D_4__Muribaculaceae;D_5__uncultured bacterium*	Diet specific y ~ sex + diet*m	UNC140463041	backupUNC14027	UNC140401510	*Asvq22*	14	74.3
Bacteroidetes	2cc64f7f2dcfc098d317347e8d3323aa	*D_4__Muribaculaceae;D_5__uncultured bacterium*	Diet specific y ~ sex + diet*m	JAX00430230	UNC170009437	backupUNC1700	*Asvq5*	17	5.6
Bacteroidetes	d6554036e1a2bd9f0586972111720879	*D_5__Rikenellaceae RC9 gut group*	Diet specific y ~ sex + diet*m	UNC160227296	UNC160235091	JAX00429228	*Asvq23*	16	72.8
Bacteroidetes	a371498f7ad6d1368940364ff67938b6	*D_5__Parabacteroides*	Diet specific y ~ sex + diet*m	UNC160105631	JAX00427104	JAX00429228	*Asvq24*	16	72.0
Firmicutes	313437e365ff76e6d19ef1ff002d7fb9	*D_5__Lactobacillus*	Diet specific y ~ sex + diet*m	UNC080206141	backupUNC08155	UNC080368796	*Asvq25*	8	43.4
Firmicutes	8ab9ece30db28dd09f0e76f426a709c9	*D_5__Streptococcus*	Diet specific y ~ sex + diet*m	JAX00165590	UNC080671076	backupUNC0803	*Asvq9*	8	102.4
Firmicutes	8ab9ece30db28dd09f0e76f426a709c9	*D_5__Streptococcus*	Diet specific y ~ sex + diet*m	UNC080206141	JAX00671644	UNC080301746	*Asvq10*	8	43.4
Firmicutes	fc35a7e21e6a077a01e39949e08917d2	*D_4__Clostridiales vadinBB60 group;D_5__uncultured bacterium*	Diet specific y ~ sex + diet*m	backupUNC0600	0JAX00137865	JAX00606563	*Asvq26*	6	4.9
Firmicutes	6f2c4135b8fa13845d9bfdcab8aafeb2	*D_4__Lachnospiraceae;D_5__uncultured*	Diet specific y ~ sex + diet*m	UNC010667356	UNC010674652	UNC010475128	*Asvq27*	1	181.1
Phylum	ASV barcode	Maximum taxonomic information	Model	Left marker	Top marker	Right marker	QTL identifier	Chr	Start (Mb)
Bacteroidetes	7738a54ecc2a9056c502e19953a34adb	*D_5__Alistipes*	Sex specific y ~ diet + sex*m	UNC130013342	backupJAX00351	4UNC130039434	*Asvq28*	13	12.5

Phylum	Top (Mb)	End (Mb)	LOD §	Effect of B6/FVB alleles §	Effect of FVB/FVB alleles §	Top marker (% variance explained)	Sex (% variance explained)	Diet (% variance explained)	Sex * Diet (% Variance Explained)
Actinobacteria	41.0	58.6	5.88^a^	− 15.95	− 30.70	5.79***	4.88***	2.37***	0.26
Actinobacteria	51.0	62.8	4.66^b^	− 4.95	18.19	4.45***	1.98**	8.59***	0.01
Bacteroidetes	76.0	83.1	4.16^b^	− 4.74	16.50	3.41***	0.07	22.92***	< 0.01
Bacteroidetes	7.1	19.6	4.97^b^	8.82	26.77	4.26***	0.2	19.09***	0.11
Bacteroidetes	7.1	19.6	4.32^b^	− 3.12	18.84	3.98***	0.05	13.67***	0.12
Bacteroidetes	105.1	116.6	5.15^b^	2.79	19.26	3.72***	1.57**	25.28***	5.04***
Bacteroidetes	180.2	193.3	4.05^b^	14.52	26.93	4.08***	1.37*	5.83***	0.11
Bacteroidetes	47.3	56.3	4.17^b^	− 17.71	− 20.93	3.57***	0.04	19.7***	0.13
Firmicutes	117.1	122.5	5.44^a^	3.49	20.68	3.14***	0.91**	43.41***	1.57***
Firmicutes	69.3	84.1	5.03^b^	11.74	20.72	2.92***	0.97**	43.19***	1.29**
Firmicutes	76.4	80.8	4.5^b^	− 18.84	− 25.37	4.52***	0.78	6.08***	0.04
Firmicutes	102.4	115.9	4.41^b^	5.60	− 18.11	4.25***	1.13*	6.65***	2.75***
Firmicutes	37.5	48.5	4.05^b^	− 18.36	− 4.08	3.38***	0.08	24.50***	0.23
Firmicutes	96.8	119.7	4.13^b^	9.48	22.51	3.67***	7.26***	5.22***	4.21***
Firmicutes	18.4	37.5	4.17^b^	− 9.90	− 23.14	3.72***	7.16***	5.22***	4.23***
Firmicutes	155.9	186.3	4.25^b^	21.51	13.00	3.98***	1.85**	10.65***	1.19*
Proteobacteria	180.2	193.3	4.13^b^	19.85	19.71	3.69***	0.15	17.47***	0.12
Proteobacteria	76.4	80.8	4.49^b^	− 15.24	− 24.35	3.97***	0.16	17.81***	0.11
Phylum	Top (Mb)	End (Mb)	LOD ⌘	Effect of American B6/FVB alleles ⌘	Effect of American FVB/FVB alleles ⌘	Top marker (% variance explained)	Sex (% variance explained)	Diet (% variance explained)	Top marker * diet (% variance explained)
Bacteroidetes	21.2	37.5	4.42^b^	− 41.61	− 22.57	2.04**	0.19	19.27***	3.72***
Bacteroidetes	19.7	37.5	4.23^b^	− 39.27	− 42.36	2.65**	0.05	13.85***	3.78***
Bacteroidetes	77.5	83.1	4.28^b^	2.04	37.54	2.42***	0.18	37.42***	2.74***
Bacteroidetes	76.0	79.3	4.25^b^	− 6.75	35.00	3.41***	0.07	22.92***	3.34***
Bacteroidetes	6.2	19.6	4.27^b^	4.24	44.19	3.79***	0.05	13.67***	3.77***
Bacteroidetes	79.6	96.5	4.03^b^	− 43.76	− 38.57	2.02**	0.8	10.28***	3.76***
Bacteroidetes	87.6	96.5	4.04^b^	− 30.20	− 51.51	0.86	0.02	3.91***	4.11***
Firmicutes	80.5	97.8	4.13^b^	24.13	52.27	0.9	2.06**	1.73**	4.22***
Firmicutes	117.1	122.5	5.98^a^	4.91	42.32	3.14***	0.91**	43.41***	3.34***
Firmicutes	69.3	84.1	4.33^b^	24.52	39.92	2.92***	0.97**	43.19***	2.45***
Firmicutes	10.5	33.5	4.33^b^	− 19.90	− 52.94	0.4	0.1	1.51**	4.56***
Firmicutes	187.2	191.6	4.71^b^	− 41.34	− 50.88	2.33**	1.15*	12.54***	4.21***
Phylum	Top (Mb)	End (Mb)	LOD ⌘	Effect of male B6/FVB alleles ⌘	Effect of male FVB/FVB alleles ⌘	Top marker (% variance explained)	Sex (% variance explained)	Diet (% variance explained)	Top marker * sex (% variance explained)
Bacteroidetes	18.4	37.5	5.72^a^	− 15.36	32.02	0.44	1.66	25.35***	4.41***

^§^ LOD and allele effects are provided for the top marker in each confidence interval

⌘ LOD and allele effects are provided for the interactive term in each confidence interval

^a^ Genome-wide significance threshold of high significance (*p* < 0.01)

^b^ Genome-wide significance threshold of significance (*p* < 0.05) *p < 0.05; ** *p* < 0.01; *** *p* < 0.001

### Metabolic phenotypes

The data analysis and collection methods for fat mass gain and serum HDL cholesterol concentration have been described previously [23]. Briefly, Echo magnetic resonance spectroscopy (MRI) (EchoMRI, Houston, TX, USA) was used to measure the fat and lean mass of all individuals. Using serum obtained from blood collected at the end of the feeding trial, total cholesterol, HDL, and LDL measurements were performed in duplicate using the EnzyChrom AF HDL and LDL/VLDL Assay kit (BioAssay Systems, Hayward, CA, USA).

### Genotyping

The genotyping analysis and collection methods have been described previously [23]. Briefly, the F2 population was genotyped on the Mouse Universal Genotyping Array (MUGA) that includes 7854 SNP markers [24]. Markers that were not polymorphic between B6 and FVB were removed from the dataset and uncertain genotype calls for individuals (GenCall score quality metric < 0.7) were set to missing. The remaining markers were used to generate a genetic map to check for problematic markers and/or sample DNAs. After all corrections, 1667 markers were used for the association analyses. Updated MUGA marker annotation was obtained from Dr. Karl Broman (https://kbroman.org/MUGAarrays/new_annotations.html).

### Statistical analyses

#### Linkage analysis

For microbiota phenotypes, a core measurable microbiota (CMM) was defined as those traits present in at least 20% of the individuals. Thresholds ranging from 0.25 to 10% have been applied for differential abundance analyses by others [25–27]. For linkage analyses, more stringent thresholds have been applied to define the CMM [28]. With this threshold, we expect to capture the anticipated 1:2:1 ratio among genotypes and most microbial traits in the F2 population. The CMM consists of 134 ASVs. After determining organisms present in the CMM, absolute microbial abundances (counts) were quantile normalized for linkage analyses. Normal quantiles were calculated with the preprocessCore R package from Dr. Ben Bolstad, version 1.46.0 (https://github.com/bmbolstad/preprocessCore).

QTL mapping was performed on metabolic and microbial features (y) in all F2 mice from both sexes and diets, and linear models using ANOVA targeted three types of genetic effects: (1) QTL effects, whereby the effect of a marker SNP is tested after controlling for sex, diet, and sex by diet interaction, which we describe in the formula as y ~ sex + diet + sex:diet + [marker], where the term in brackets is the alternative but not the null model; (2) QTL by diet effects, using y ~ sex + diet + marker + [diet:marker]; and (3); QTL by sex effects, using y ~ sex + diet + marker + [sex:marker]. QTL peaks with a logarithm of the odds (LOD) greater than thresholds determined by 10,000 permutations were considered genome-wide significant (*p* < 0.05, LOD > 4.00 microbial abundance, 3.90 measures of diversity) or highly significant (*p* < 0.01, LOD > 5.19 microbial abundance, 4.68 measures of diversity) for all models. The thresholds applied to microbial abundance and measures of diversity reflect the average genome-wide significant thresholds for all ASV present within the CMM and for all measures of diversity analyzed respectively. A LOD drop of 1.5 LOD from the top marker was used to determine the 95% confidence intervals for each QTL. Linear models using ANOVA were used to check for any interactions between sex and/or diet with the top markers of each QTL. The variance explained by the top markers at each QTL in the combined model was calculated by dividing the sum of squares of the model including the top marker by the total sum of squares of the model without QTL. The variance explained by the top markers at each QTL in the interactive models was calculated by dividing the sum of squares of the model including the interaction between diet and the top marker or sex and the top marker by the total sum of squares of the model without QTL.

Several limitations exist in the available literature for microbiome QTL analysis including the current work. Microbial data is zero-inflated compositional data [29] and to-date no appropriate statistical method has been developed to transform and perform QTL analysis that fully addresses zero-inflation and compositional nature of the data. Zero-inflation might be the result of true biological variation or technical variation in current technologies for measuring abundances of organisms [30, 31]. Of note, normalizing quantiles does not force the data into a normal distribution but rather, makes the individual microbial features more similar in statistical properties. Classical approaches to data transformation, for example, the log transformation on individual ASV, similarly fail to achieve a normal distribution and otherwise substantially alter the distribution of only the non-zero data (zero data cannot be transformed). Another common approach to overcome these limitations is binary modeling of the presence or absence of specific organisms followed by linear modeling of specific organisms for only hosts identified with non-zero counts of that organism. However, this two-step approach is limited to single predictors and reduces power without addressing the zero-inflated and compositional nature of the data encountered in the one-step procedure. After careful consideration of these alternatives, we chose to use a combination of permutation and preprocessCore’s quantile normalization across all ASV present in the CMM. Forcing the skewed data into the same distribution means that the data will behave similarly under permutation which allowed us to determine how unusual specific LOD scores were in the permutated data to identify appropriate thresholds of significance. We acknowledge a great deal of variation in methods used to normalize microbial features and even in the definition of the CMM as well as the great need to standardize these methods between investigators. Continued growth of statistical methods for zero-inflated compositional microbial data sets is needed. Our implementation has been made publicly available at https://github.com/annacsalvador/Salvador_Microbiome_2023.

#### Confirmatory factor analysis and structural equation modeling

Confirmatory factor analysis (CFA) was conducted with the lavaan R package for structural equation modeling (SEM) from Dr. Yves Rosseel, version 0.6.9 (https://www.jstatsoft.org/index.php/jss/article/view/v048i02/2448) [32]. Initial models were selected based on information from individual QTL models and correlations among traits within and between QTL models. All traits were collapsed into four, ordinal quantiles for CFA and diagonally weighted least squares estimator was used based on methods described elsewhere [33]. The final structural models illustrate QTL models for overlapping microbial and metabolic traits and are refined to include only predictors for which pathway coefficients are significantly different from zero, indicating that each of the remaining predictors in the model is significantly associating with one or more endogenous or exogenous variable. Our implementation has been made publicly available at https://github.com/annacsalvador/Salvador_Microbiome_2023.

### Candidate gene annotation

All genes within each significant QTL confidence interval were annotated with KEGG pathway identifiers. Candidate genes were furthered characterized by KEGG pathways related to glucose, insulin, fatty acids, adipocytes, cholesterol, obesity, diabetes mellitus, metabolic syndrome, digestion and absorption of carbohydrates, fats, and proteins, the epithelial barrier, and the immune system. A comprehensive list of KEGG pathway queries is provided in Supplementary Table S3. Transcript variants between the parental mouse strains were identified in genes annotated for selected KEGG pathways of interest from the Mouse Genome Informatics Strains, SNPS, and Polymorphisms database. Tissue-specific expression was determined with Mouse ENCODE Transcriptome data, accessed through the National Library of Medicine National Center for Biotechnology database.

## Results

### Diet is a strong modulator of the gut microbiome

**Fig. 1:**
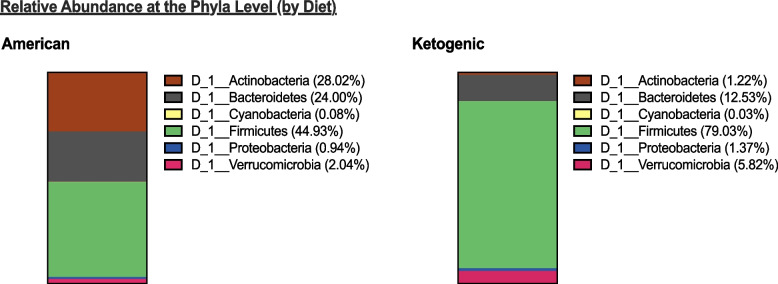
Relative abundance of microbiota and correlations at the phyla level (by diet). Relative abundance of Firmicutes in F2s on the ketogenic diet is nearly twice as high as the relative abundance of Firmicutes in F2s on the American diet at the expense of Actinobacteria, and Bacteroidetes for which the relative abundances are lower in F2s on the ketogenic diet

Diet explains a large proportion of variation in the abundance of microbiota at the Phyla level irrespective to genetic background. Diet explains 64.79% of variation in abundance of Actinobacteria, 61.22% of variation in the abundance of Firmicutes, and 25.49% of variation in the abundance of Bacteroidetes (Supplementary Table S4). The relative abundance of Firmicutes in F2 on the ketogenic diet is nearly twice as high as in F2 on the American diet (Fig. 1). This increase in Firmicutes in F2 on the ketogenic diet appears to occur at the expense of the relative abundance of Actinobacteria and Bacteroidetes (Fig. 1).

**Fig. 2:**
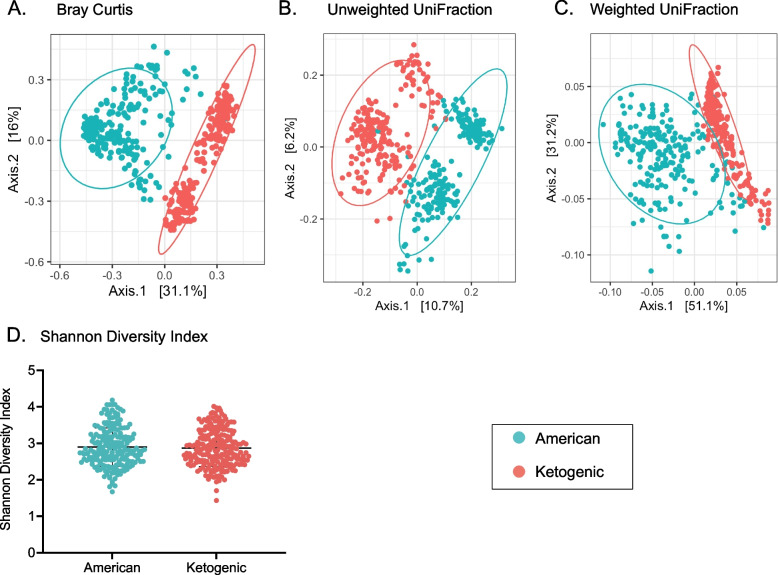
Beta diversity Principal Coordinate Analysis (PCoA). **A** Bray Curtis Index. PC1 and PC2 describe 31.1% and 16% of the variation in ASV respectively. **B** Unweighted UniFraction. PC1 and PC2 describe 10.7% and 6.2% of the variation in ASV respectively. **C** Weighted UniFraction. PC1 and PC2 describe 51.1% and 31.2% of the variation in ASV respectively. Overlaying diet with the measures of beta diversity illustrates two distinct groups, roughly segregating PC1 for all measures of beta diversity. **D** Shannon Diversity Index. The Shannon Diversity Index is not different between diet groups

Principal coordinate analysis (PCoA) for measures of beta diversity revealed two distinct groups segregating at PC1. The Bray–Curtis index PCo1 and PCo2 describe 31.1% and 16% of the variation in ASV respectively (Fig. 2A). The Jaccard Index PCo1 and PCo2 are nearly identical to the Bray–Curtis index and describe 21.3% and 10.6% of variation in ASV respectively (Supplementary Fig. S2). Unweighted UniFraction PCo1 and PCo2 describe 10.7% and 6.2% of variation in ASV respectively (Fig. 2B). Weighted UniFraction PCo1 and PCo2 describe 51.1% and 31.2% of variation in ASV respectively (Fig. 2C). Overlaying diet with the data illustrates two distinct groups roughly segregating PCo1 for all measures of beta diversity. However, alpha diversity, illustrated by the Shannon Diversity Index, does not depend on diet (Fig. 2D). Eigenvectors extracted from the PCo1 and PCo2 from the Bray–Curtis index, Jaccard index, unweighted and weighted UniFractions, as well as values from the Shannon Diversity Index served as additional traits for linkage analysis below.

### Microbial features are modulated by genetic loci

**Fig. 3:**
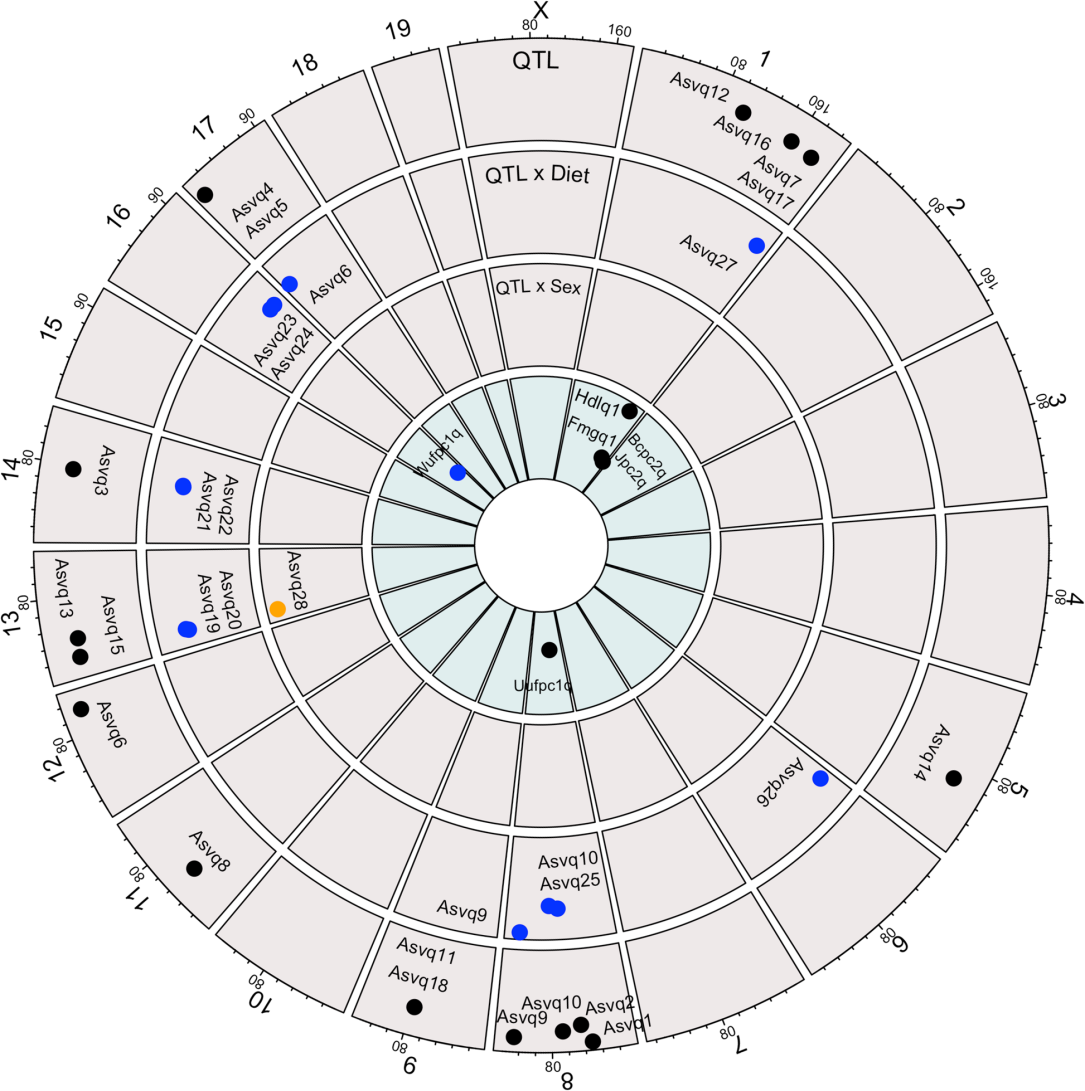
Microbial QTL. Outer ring to inner ring: Significant QTL in the QTL model (black) for ASV associated with the genus *Coriobacteriaceae UCG-002* on Chr 8 at *Asvq1*, the genus *Enterohabdus* on Chr 8 at *Asvq2*, multiple uncultured bacterium from the *Muribaculaceae* family on Chr 14 at *Asvq3* and Chr 17 at *Asvq4* and *Asvq5*, the genus *Alistipes* on Chr 12 at *Asvq6,* the genus *Rikenella* on Chr 1 at *Asvq7*, the genus *Rikenellaceae RC9 Gut Group* on Chr 11 at *Asvq8*, the genus *Streptococcus* on Chr 8 at *Asvq9* and *Asvq10*, the genus *GCA-900066575* on Chr 9 at *Asvq11*, the genus *Lachnoclostridium* on Chr 1 at *Asvq12* and Chr 13 at *Asvq13*, the genus *Romboutsia* on Chr 5 at *Asvq14* and Chr 13 at *Asvq15*, the genus *Ruminiclostridium 9* on Chr 1 at *Asvq16*, and the genus *Bilophila* on Chr 1 at *Asvq17* and Chr 9 at *Asvq18*. Significant QTL in the diet specific model (blue) for ASV associated with multiple uncultured bacterium from the *Muribaculaceae* family on Chr 13 at *Asvq19* and *Asvq20*, Chr 14 at *Asvq21* and *Asvq22*, and Chr 17 at *Asvq5*, the genus *Rikenelleceae RC9 Gut Group* on Chr 16 at *Asvq23*, the genus *Parabacteroides* on Chr 16 at *Asvq24*, the genus *Lactobacillus* on Chr 8 at *Asvq25*, multiple ASV from the *Streptococcus* genus on Chr 8 at *Asvq9*, and *Asvq10*, the uncultured genera from the family of *Clostridiales vadinBB60 group* on Chr 6 at *Asvq26*, and the *Lachnospiraceae* family on Chr 1 at *Asvq27*. A single significant QTL in the sex specific model (orange) for the genus *Alistipes* on Chr 13 at *Asvq28*; Previously identified QTL for metabolic traits and diversity measures in the combined model (black) and diet specific model (blue). Fat mass gain during the feeding trial on Chr 1 at *Fmgq1*, along with serum HDL cholesterol concentration after the feeding trial on Chr 1 at *Hdlq1*, Bray–Curtis and Jaccard measures of beta diversity at *Bcpc2q* and *Jpc2q*. Unweighted unifraction on Chr 8 at *Uufpc2q*, and weighted unifraction on Chr 16 at *Wufpc1q*. *Fmgq1*, *Hdlq1*, *Bcpc2q*, and *Jpc2q* overlap the same region of the genome as *Asvq7*, *Asvq16*, and *Asvq17* for uncultured *Rikenella*, *Ruminiclostridium*, and uncultured *Bilophila*. *Wufpc1q* overlaps the same region of the genome for *Asvq23* and *Asvq24* for *Rikenelleceae RC9 gut group* and *Parabacteroides*

In the sex and diet-independent QTL model, which tests for marginal effect QTL after controlling for sex and diet (see the “Methods” section; y ~ sex + diet + sex:diet + [marker]), 18 distinct QTL were detected for 15 unique microbial abundances (counts), there were 119 additional organisms remaining within the CMM that did not display a genetic linkage (Fig. 3, Table 1).

*Asvq7*, for the genus *Rikenella* overlaps with *Asvq16* and *Asvq17* for *Ruminiclostridium 9* and *Bilophila* genera, respectively, as well as with the previously identified QTL for fat mass gain (*Fmgq1*) and serum HDL cholesterol concentration (*Hdlq1*).

Apart from *Coriobacteriaceae* (*Asvq1*), diet appears to explain a more significant proportion of the variation in the abundance of these ASV despite this QTL not being diet specific. This is the only ASV with a QTL for which the top marker explains a greater proportion of the variation in the abundance of the organism than diet itself despite there being a significant effect of diet as well in this model (Table 1). For all the remaining loci identified under the QTL model, diet explains a greater proportion of the variation than the top marker does at each QTL. This is particularly clear for *Streptococcus* where diet explains nearly 50% of the variation in abundance of two *Streptococcus* ASVs while the top markers at *Asvq9* and *Asvq10* explain only 3.14% and 2.92% of the variation, respectively (Table 1).

### Microbial features are modulated by diet-specific genetic loci

The analysis for the interaction between QTL and diet detected 12 QTL for 11 unique microbial features, there were 123 organisms remaining within the CMM not displaying diet-specific genetic linkage (Fig. 3, Table 1). Nine of these QTLs were distinct from the ones identified in the QTL model.

Of note, three diet-specific QTLs were identified that are identical or nearly identical to *Asvq5*,* Asvq9*, and *Asvq10* that were identified in the QTL model for the same organisms. There are only modest differences in the 95% confidence intervals at *Asvq9* and *Asvq10* in the loci identified in the QTL and QTL by diet models. The top marker is unchanged between the QTL and QTL by diet models at all 3 loci so these loci will continue to be referred to as *Asvq5*, *Asvq9*, and *Asvq10*.

Interestingly, diet alone explains a greater proportion of the variation than the interaction between diet and the top marker at the diet-specific QTL for *Muribaculeceae* (*Asvq19*,* Asvq20*,* Asvq21*,* Asvq22*,* Asvq5*),* Rikenellaceae RC9* Gut Group (*Asvq23*),* Streptococcus* (*Asvq9*,* Asvq10*), and the uncultured geneus of the *Lachnospiraceae* family (*Asvq27*) (Table 1). The interaction between genotype and diet appears to explain the greatest proportion of variation at *Asvq24* for *Parabacteroides* (4.11%), *Asvq25* for *Lactobacillus* (4.22%), and *Asvq26* for *Clostridiales* VadinBB60 Group (4.56%) (Table 1).

### Microbial features are modulated by sex-specific genetic loci

The QTL by sex analysis detected a single significant QTL for *Alistipes* on Chr 13 at 18.4 Mb (*Asvq28*) (Fig. 3, Table 1).

Sex explains 1.66% of variation in the abundance of *Alistipes* while the interaction between sex and the top marker at *Asvq28* explains over 4% of variation in the abundance of this OTU (Table 1). Even in the sex-specific model of *Asvq28*, diet explains a greater proportion of the variation than either sex or the interaction between sex and the genotype.

### Measures of beta diversity are genetically modulated

In the QTL model, three distinct QTL were identified for PCo2 of several measures of beta diversity including Bray–Curtis, Jaccard, and the unweighted UniFraction (Fig. 3, Table 2). The eigenvectors for both Bray–Curtis and the Jaccard index PCo2 map to Chr 1 at 177.5 Mb (*Bcpc2q*; *p* < 0.05, CI = 160.6–185.1 Mb; *Jpc2q*; *p* < 0.05, CI = 160.6 = 185.1 Mb), overlapping with *Asvq7* (151.9–193.3 Mb), *Asvq16* (138–186.3 Mb), *Asvq17* (144.5–193.3 Mb) and previously identified QTL for fat mass gain (*Fmgq1*; 180–194.4 Mb) and serum HDL cholesterol concentration (*Hdlq1*; 160.6–176.1 Mb) [23]. As noted above, PCo2 of Bray–Curtis and the Jaccard index appears to be modestly represented by alpha diversity. The remaining locus identified by the QTL model was for unweighted UniFraction PC2 at 53.7 Mb (*Uwufpc2q*; *p* < 0.05, CI = 43.4–62.8 Mb). No QTL were identified for the Shannon Diversity Index.

**Table 2:** Linkage analysis for microbial metadata

Phenotype	Model	Left marker	Top marker	Right marker	QTL identifier	Chr	Start (Mb)	Top (Mb)	End (Mb)	LOD §	Effect of B6/FVB alleles §	Effect of FVB/FVB alleles §	Top marker (% variance explained)	Sex (% variance explained)	Diet (% variance explained)	Sex * diet (% variance explained)
Bray Curtis PCo2	Combined y ~ m + sex*diet	UNC010637850	backupJAX00277549	UNC010414123	*Bcpc2q*	1	160.6	177.5	185.1	4.39^b^	− 0.10	− 0.10	4.16***	0.15	10.18***	0.83*
Jaccard PCo2	Combined y ~ m + sex*diet	UNC010637850	backupJAX00277549	UNC010414123	*Jpc2q*	1	160.6	177.5	185.1	4.12^b^	− 0.08	− 0.09	3.86***	0.12	11.65***	0.58
Unweighted UniFraction PCo2	Combined y ~ m + sex*diet	UNC080206141	UNC080229115	UNC080591069	*Uufpc2q*	8	43.4	53.7	62.8	4.16^b^	− 0.03	0.02	3.58***	< 0.01	15.48***	4.17***
Phenotype	Model	Left marker	Top marker	Right marker	QTL identifier	Chr	Start (Mb)	Top (Mb)	End (Mb)	LOD ⌘	Effect of American B6/FVB alleles ⌘	Effect of American FVB/FVB alleles ⌘	Top marker (% variance explained)	Sex (% variance explained)	Diet (% variance explained)	Top marker * diet (% variance explained)
Weighted UniFraction PCo1	Diet specific y ~ sex + diet*m	UNC160227296	UNC160235091	UNC160983056	*Wufpc1q*	16	72.8	79.6	95.8	4.97^a^	− 0.03	− 0.03	1.14**	0.01	59.26***	2.1***

^§^ LOD and allele effects are provided for the top marker in each confidence interval

⌘ LOD and allele effects are provided for the interactive term in each confidence interval

^a^ Genome-wide significance threshold of high significance (*p* < 0.01)

^b^ Genome-wide significance threshold of significance (*p* < 0.05) * *p* < 0.05; ** *p* < 0.01; *** *p* < 0.001

In the QTL by diet analysis, one additional QTL was identified for weighted UniFraction PC1 on Chr 16 at 79.6 Mb (*Wufpc1q*; *p* < 0.01, 95% CI = 72.8–95.8 Mb), overlapping with *Asvq23* and *Asvq24* for the *Rikenelleceae RC9* Gut Group and *Parabacteroides* (*Asvq23*; 72.8–96.5 Mb, *Asvq24*; 72–96.5 Mb).

### Candidate keystone species modulating the microbiome and physiological traits

**Fig. 4:**
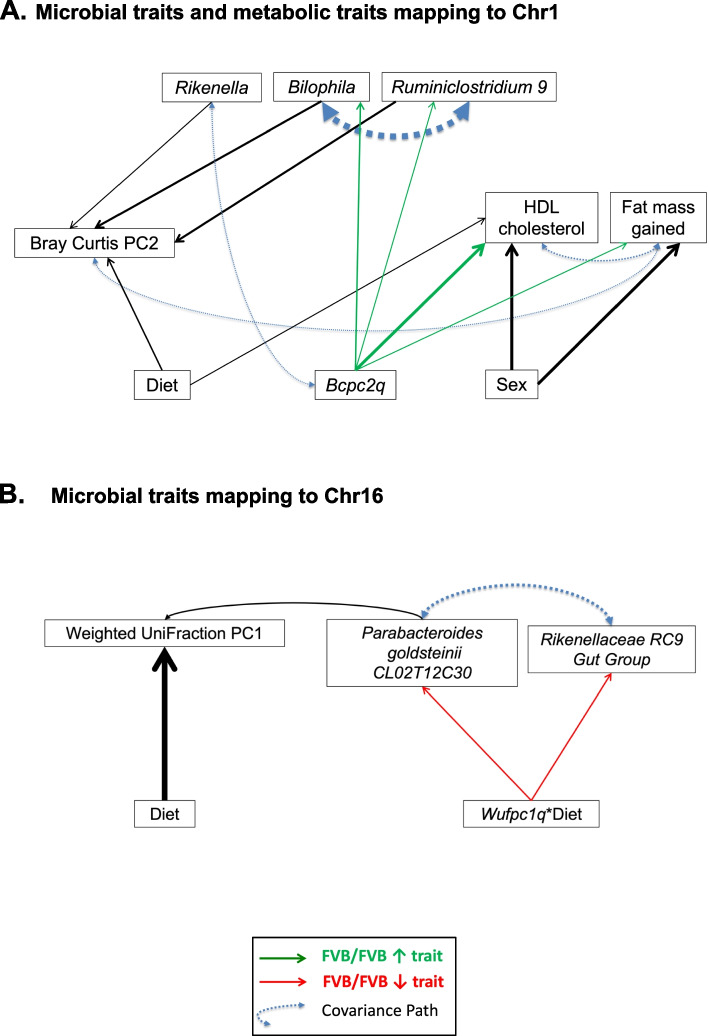
Graphical representation of SEM. Solid, single-headed arrows indicate the direction of paths and the weight of each arrow is proportional to the path coefficient (r) from the predictor to the variable and the percentage of variation in the variable that is explained by each predictor. Positive effects (green arrows) indicate that the FVB allele increases the trait; negative effects (red arrows) indicate that the FVB allele decreases the trait. Double-headed, blue arrows represent covariate pathways detected in the structural model of microbial features and measures of diversity

A structural equation model (SEM) was built to illustrate the magnitude of the effects of each predictor in the models of *Bilophila*,* Rikenella*,* Ruminiclostridium 9*, and Bray–Curtis PCo2, all mapping to the distal part of Chr 1 (Fig. 4A). Genotypes at *Bcpc2q* were chosen to model all traits mapping to the distal part of Chr 1 as *Bcpc2q* is contained inside of the confidence interval for the other three loci mapping to the distal region of Chr 1. The initial model was refined until path coefficients were all significantly different from zero, suggesting that each of the remaining predictors in the model is significantly associating with one or more of the other predictors (Table 3). The refined model suggests that the FVB/FVB genotype at *Bcpc2q* increases abundances of *Bilophila*,* Ruminiclostridium 9*, and *Rikenella*, and these three ASV are driving differences in the Bray–Curtis index PCo2 (Fig. 4A). A covariance pathway is detected among abundances of *Bilophila* and *Ruminiclostridium 9* in addition to their individual, direct effects on the Bray–Curtis index. The inclusion of metabolic traits does not elucidate direct, indirect, or covariance pathways between the metabolic traits and specific organisms mapping to distal Chr 1. However, a covariance pathway is observed between the Bray–Curtis index PCo2 and the amount of fat mass gained.

**Table 3:** Conditioned linkage analysis and structural modeling of overlapping QTL

Confirmatory factor analysis and structural models			
Variable	Predictor	Path coefficient (r)	*t*-statistic of path coefficient
Bray Curtis PC2	Diet	0.25	< 0.001
	ecfbbfd673f8a3e1c34718d968e6b069 (*Rikenella*)	− 0.17	0.002
	b89e2f23a606787577644904f2b6aed3 (*Ruminiclostridium 9*)	− 0.35	< 0.001
	X18f60eae2c3a26bab8efa0f9823e31b4 (*Bilophila*)	− 0.24	0.014
X18f60eae2c3a26bab8efa0f9823e31b4 (*Bilophila*)	*Bcpc2q*	0.26	< 0.001
b89e2f23a606787577644904f2b6aed3 (*Ruminiclostridium 9*)	*Bcpc2q*	0.14	0.003
Serum HDL cholesterol concentration (ng/mL)	Sex	0.48	< 0.001
	Diet	0.19	0.003
	*Bcpc2q*	0.40	< 0.001

Similarly, a SEM was built to illustrate the magnitude of effects of each predictor in the models of *Parabacteroides*, *Rikenellaceae RC9 gut group*, and weighted UniFraction PCo1, all mapping to distal Chr 16 (Fig. 4B). Genotypes at *Wufpc1q* were chosen for the model as the 95% confidence interval for this locus is contained within the QTL of all other traits in this structural model. The path coefficients are again, all significantly different from zero (Table 3). The refined model suggests that the interaction between the FVB/FVB genotype and the American diet at *Wufpc1q* directly decrease abundances of *Parabacteroides* and the *Rikenelleceae RC9 Gut Group*. The abundance of *Parabacteroides* has a direct relationship with the weighted UniFraction PCo1. A covariance pathway is detected among abundances of *Parabacteroides* and the *Rikenelleceae RC9 Gut Group.*

### Identification of candidate genes at Asvq7, Asvq16, Asvq17, and Bcpc2q

Candidate genes that might elucidate the relationship between *Rikenella*, *Ruminiclostridium 9*, *Bilophila*, and the Bray–Curtis PCo2 were investigated. *Bcpc2q* is contained inside of the confidence interval for the other three loci mapping to the distal region of Chr 1. Positional candidates at *Bcpc2q* that overlap with one or more metabolic KEGG pathways are summarized in Table 4. Out of 275 positional candidates at *Bcpc2q*, 35 genes overlap with one or more KEGG pathways. Eleven out of the total 35 positional candidates harbor known non-synonymous transcript variants diverging between these strains. The presence of these non-synonymous transcript variants makes *Aim2*, *Apoa2*, *Atp1a4*, *Cadm3*, *Cd244a*, *Cd48*, *F11r*, *Fcer1g*, *Mpz*, *Ndufs2*, *Sdhc*, and *Sell* the primary candidate genes of interest in this region. Of these, *F11r*, *Fcer1g*, *Ndufs2* and *Sdhc* are expressed in the intestine.

**Table 4:** KEGG Pathway annotation of positional candidate genes at *Mtq7*,* Mtq8*,* Mtq13*,* Mtq14*, and* Pc1q*

QTL	Model	Phenotype	Gene symbol (MGI)	KEGG pathway
*Bcpc2q*	Combined y ~ m + sex*diet	Bray Curtis PC2	*Aim2*	Cytosolic DNA-sensing pathway (mmu04623)
			*Akt3*	Adipocytokine signaling pathway (mmu04920)
			*Akt3*	AMPK signaling pathway (mmu04152)
			*Akt3*	Carbohydrate digestion and absorption (mmu04973)
			*Akt3*	Chemokine signaling pathway (mmu04062)
			*Akt3*	Epstein-Barr virus infection (mmu05169)
			*Akt3*	Hepatitis B (mmu05161)
			*Akt3*	Hepatitis C (mmu05160)
			*Akt3*	HIF-1 signaling pathway (mmu04066)
			*Akt3*	Influenza A (mmu05164)
			*Akt3*	Insulin resistance (mmu04931)
			*Akt3*	Insulin signaling pathway (mmu04910)
			*Akt3*	Measles (mmu05162)
			*Akt3*	Non-alcoholic fatty liver disease (NAFLD) (mmu04932)
			*Akt3*	Osteoclast differentiation (mmu04380)
			*Akt3*	Prolactin signaling pathway (mmu04917)
			*Akt3*	Regulation of lipolysis in adipocytes (mmu04923)
			*Akt3*	T cell receptor signaling pathway (mmu04660)
			*Akt3*	Tight junction (mmu04530)
			*Akt3*	Toll-like receptor signaling pathway (mmu04620)
			*Akt3*	Tuberculosis (mmu05152)
			*Aldh9a1*	Glycolysis/gluconeogenesis (mmu00010)
			*Alyref2*	Herpes simplex virus 1 infection A (mmu05168)
			*Apoa2*	PPAR signaling pathway (mmu03320)
			*Atp1a2*	Bile secretion (mmu04976)
			*Atp1a2*	Carbohydrate digestion and absorption (mmu04973)
			*Atp1a2*	Mineral absorption (mmu04978)
			*Atp1a2*	Protein digestion and absorption (mmu04974)
			*Atp1a4*	Bile secretion (mmu04976)
			*Atp1a4*	Carbohydrate digestion and absorption (mmu04973)
			*Atp1a4*	Mineral absorption (mmu04978)
			*Atp1a4*	Protein digestion and absorption (mmu04974)
			*Atp1b1*	Bile secretion (mmu04976)
			*Atp1b1*	Carbohydrate digestion and absorption (mmu04973)
			*Atp1b1*	Mineral absorption (mmu04978)
			*Atp1b1*	Protein digestion and absorption (mmu04974)
			*Cadm3*	Cell adhesion molecules (mmu04514)
			*Cd244a*	Natural killer cell-mediated cytotoxicity (mmu04650)
			*Cd247*	Natural killer cell-mediated cytotoxicity (mmu04650)
			*Cd247*	T cell receptor signaling pathway (mmu04660)
			*Cd48*	Natural killer cell-mediated cytotoxicity (mmu04650)
			*Ephx1*	Bile secretion (mmu04976)
			*F11r*	Cell adhesion molecules (mmu04514)
			*F11r*	Leukocyte transendothelial migration (mmu04670)
			*F11r*	Tight junction (mmu04530)
			*Fasl*	African trypanosomiasis (mmu05143)
			*Fasl*	Allograft rejection (mmu05330)
			*Fasl*	Autoimmune thyroid disease (mmu05320)
			*Fasl*	Hepatitis B (mmu05161)
			*Fasl*	Herpes simplex virus 1 infection A (mmu05168)
			*Fasl*	Influenza A (mmu05164)
			*Fasl*	Measles (mmu05162)
			*Fasl*	Natural killer cell-mediated cytotoxicity (mmu04650)
			*Fasl*	Non-alcoholic fatty liver disease (NAFLD) (mmu04932)
			*Fasl*	Type I diabetes mellitus (mmu04940)
			*Fcer1g*	Natural killer cell-mediated cytotoxicity (mmu04650)
			*Fcer1g*	Tuberculosis (mmu05152)
			*Fcgr2b*	Measles (mmu05162)
			*Fcgr2b*	Osteoclast differentiation (mmu04380)
			*Fcgr2b*	Staphylococcus aureus infection (mmu05150)
			*Fcgr2b*	Tuberculosis (mmu05152)
			*Fcgr3*	Leishmaniasis (mmu05140)
			*Fcgr3*	Osteoclast differentiation (mmu04380)
			*Fcgr3*	Staphylococcus aureus infection (mmu05150)
			*Fcgr3*	Tuberculosis (mmu05152)
			*Fcgr4*	Leishmaniasis (mmu05140)
			*Fcgr4*	Natural killer cell-mediated cytotoxicity (mmu04650)
			*Fcgr4*	Osteoclast differentiation (mmu04380)
			*Fcgr4*	Staphylococcus aureus infection (mmu05150)
			*Fcgr4*	Systemic lupus erythematosus (mmu05322)
			*Fcgr4*	Tuberculosis (mmu05152)
			*H3f3a*	Systemic lupus erythematosus (mmu05322)
			*Hsd17b7*	Ovarian steroidogenesis (mmu04913)
			*Hsd17b7*	Steroid biosynthesis (mmu00100)
			*Hsd17b7*	Steroid hormone biosynthesis (mmu00140)
			*Ifi202b*	Cytosolic DNA-sensing pathway (mmu04623)
			*Mpz*	Cell adhesion molecules (mmu04514)
			*Mpzl1*	Cell adhesion molecules (mmu04514)
			*Ndufs2*	Non-alcoholic fatty liver disease (NAFLD) (mmu04932)
			*Pex19*	Peroxisome (mmu04146)
			*Rxrg*	Adipocytokine signaling pathway (mmu04920)
			*Rxrg*	PPAR signaling pathway (mmu03320)
			*Sdhc*	Non-alcoholic fatty liver disease (NAFLD) (mmu04932)
			*Sele*	African trypanosomiasis (mmu05143)
			*Sele*	Cell adhesion molecules (mmu04514)
			*Sell*	Cell adhesion molecules (mmu04514)
			*Selp*	Cell adhesion molecules (mmu04514)
			*Selp*	Staphylococcus aureus infection (mmu05150)
			*Sh2d1b1*	Natural killer cell-mediated cytotoxicity (mmu04650)
			*Slamf1*	Measles (mmu05162)
			*Tlr5*	Inflammatory bowel disease (mmu05321)
			*Tlr5*	Salmonella infection (mmu05132)
			*Tlr5*	Toll-like receptor signaling pathway (mmu04620)
			*Xcl1*	Chemokine signaling pathway (mmu04062)
*Wufpc1q*	Diet specific y ~ sex + diet*m	Weighted UniFraction PC1	*Cldn14*	Cell adhesion molecules (mmu04514)
			*Cldn14*	Hepatitis C (mmu05160)
			*Cldn14*	Leukocyte transendothelial migration (mmu04670)
			*Cldn14*	Tight junction (mmu04530)
			*Cldn17*	Cell adhesion molecules (mmu04514)
			*Cldn17*	Hepatitis C (mmu05160)
			*Cldn17*	Leukocyte transendothelial migration (mmu04670)
			*Cldn17*	Tight junction (mmu04530)
			*Cldn8*	Cell adhesion molecules (mmu04514)
			*Cldn8*	Hepatitis C (mmu05160)
			*Cldn8*	Leukocyte transendothelial migration (mmu04670)
			*Cldn8*	Tight junction (mmu04530)
			*Cxadr*	Viral myocarditis (mmu05416)
			*Ifnar1*	Hepatitis B (mmu05161)
			*Ifnar1*	Hepatitis C (mmu05160)
			*Ifnar1*	Herpes simplex virus 1 infection A (mmu05168)
			*Ifnar1*	Influenza A (mmu05164)
			*Ifnar1*	Measles (mmu05162)
			*Ifnar1*	Natural killer cell-mediated cytotoxicity (mmu04650)
			*Ifnar1*	Osteoclast differentiation (mmu04380)
			*Ifnar1*	Toll-like receptor signaling pathway (mmu04620)
			*Ifnar2*	Hepatitis C (mmu05160)
			*Ifnar2*	Herpes simplex virus 1 infection A (mmu05168)
			*Ifnar2*	Influenza A (mmu05164)
			*Ifnar2*	Measles (mmu05162)
			*Ifnar2*	Natural killer cell-mediated cytotoxicity (mmu04650)
			*Ifnar2*	Osteoclast differentiation (mmu04380)
			*Ifnar2*	Toll-like receptor signaling pathway (mmu04620)
			*Ifngr2*	Herpes simplex virus 1 infection A (mmu05168)
			*Ifngr2*	HIF-1 signaling pathway (mmu04066)
			*Ifngr2*	Inflammatory bowel disease (mmu05321)
			*Ifngr2*	Influenza A (mmu05164)
			*Ifngr2*	Leishmaniasis (mmu05140)
			*Ifngr2*	Measles (mmu05162)
			*Ifngr2*	Natural killer cell-mediated cytotoxicity (mmu04650)
			*Ifngr2*	Osteoclast differentiation (mmu04380)
			*Ifngr2*	Salmonella infection (mmu05132)
			*Ifngr2*	Tuberculosis (mmu05152)
			*Il10rb*	Epstein-Barr virus infection (mmu05169)
			*Il10rb*	Tuberculosis (mmu05152)
			*Jam2*	Cell adhesion molecules (mmu04514)
			*Jam2*	Leukocyte transendothelial migration (mmu04670)
			*Jam2*	Tight junction (mmu04530)
			*Ncam2*	Cell adhesion molecules (mmu04514)
			*Sod1*	Peroxisome (mmu04146)
			*Tiam1*	Chemokine signaling pathway (mmu04062)

### Identification of candidate genes at Asvq23, Asvq24, and Wufpc1q

Candidate genes were investigated that might elucidate the relationship between *Rikenelleceae RC9* Gut Group, *Parabacteroides*, weighted UniFraction, *Asvq23*,* Asvq24*, and *Wufpc1q*. Positional candidates at *Wufpc1q that* overlap with one or more metabolic KEGG pathways are summarized in Table 4. Out of 133 positional candidates at *Wufpc1q* 11 genes overlap with one or more metabolic KEGG pathways. However, none of the genes annotated with KEGG pathway from our query harbor known non-synonymous transcript variants diverging between the two strains.

## Discussion

This study provides evidence that abundances of gut microbiota are driven by unique combinations of effects from the host’s genetics, response to high fat diets varied in carbohydrate content, and sex. Many previous studies have compared the effects of control mouse diets to high fat diets where one or two representative ingredients contribute to the total fat, carbohydrate, and protein content of the diet [6]. The American and ketogenic diets used here more accurately recapitulate the diversity of ingredients found in human diets not only in terms of the macronutrient profile of human diets but also the fiber content and lipid profiles [6]. The diverse set of ingredients is particularly important to studies of the microbiota because of differences in substrate utilization between bacterial taxa. There is an abundance of literature supporting the potent effects of diet on the abundance of gut microbiota driven by fiber, carbohydrate, protein, and lipid *source* and *composition* [9, 11, 12, 16–18, 34–36]. Other studies have demonstrated that the effect of abnormal diets on gut microbiota might stifle the underlying effect of single gene mutations because diets are such a potent regulator of microbial abundances [16, 37]. These authors have called for further study of diets varied in macronutrient content and study of more complex genetic models. The current study has demonstrated that high fat diets varied in carbohydrate content continue to be commanding predictors of abundances of gut microbial abundances even in a more complex genetic model. We will highlight below many results for which genetic effects are likely dependent on specific ingredients in one of the two diets and discuss the importance of incorporating human-comparable diets into microbial studies.

Another unique feature of the current study is the incorporation of latent variables harbored in the PCoA of ASV data. Latent variables are those that are not directly observable in a model but can be inferred from other variables and can hold important information for interpreting biological relationships. PCoA of ASV data revealed that PCo1 captures the variance in ASV caused by diet while it was less clear what variance was captured by PCo2. Extraction of eigenvectors from these principal components is one way to incorporate information from the latent variables contained in the PCoA.

In this study, 18 loci diet and sex-independent loci were identified. Out of these loci, *Coriobacteriaceae* is the least influenced by diet. *Coriobacteriaceae* is the only ASV for which the top marker explains a greater proportion of the variation in the abundance of the organism than diet, suggesting that there is a genetic predisposition to having higher or lower abundances of *Coriobacteriaceae*. *Coriobacteriaceae* has previously been associated with host genetics and QTL regulating immune function and susceptibility to carcinoma and tumor development in mice [38, 39]. *Coriobacteriaceae* has been described as a dominant species in the mammalian gut and it is positively correlated with hepatic triglyceride concentration and non-HDL cholesterol concentration in mice [40].

A significant proportion of the variation in all other ASV with loci detected by the diet and sex-independent model is still driven by diet, especially for *Streptococcus* at *Asvq9* and *Asvq10*. *Streptococcus* belongs to the Firmicutes phylum. Fiber is a particularly important dietary component for modulating abundance of Firmicutes. When animals switch from a low fat/fiber rich plant diet to a high fat/high sugar diet, they experience a significant increase in the Firmicutes phylum along with a decrease in Bacteroidetes [17]. Dramatic shifts were observed in these phyla between American and ketogenic diet F2s irrespective of their genetic backgrounds. Our ketogenic diet is composed of twice as much soluble and insoluble fiber as the American diet, and this likely drives many of the differences in the abundance of OTUs from these phyla. The relative abundance of Firmicutes in F2s exposed to the ketogenic diet is nearly twice as high as F2s exposed to the ketogenic diet. It appears that this increase in Firmicutes in the F2s exposed to the ketogenic diet coincides with a decrease in the relative abundance of Bacteroidetes. Limited evidence suggests that a higher Firmicutes to Bacteroidetes ratio is positively correlated with obesity while a decrease in this ratio has been associated with inflammatory bowel disease [41]. However, controversy surrounds the association of the Firmicutes to Bacteroidetes ratio and health status [41].

Six diet-specific QTL were also identified under the QTL by diet model. Of note, all six diet-specific QTL are for microbial features from either the Firmicutes or Bacteroidetes phyla. These QTL include, *Asvq19*,* Asvq20*,* Asvq21*,* Asvq22*, and *Asvq5* for *Muribaculaceae* (Bacteroidetes),* Asvq23* for *Rikenelleceae RC9 Gut Group* (Bacteroidetes), *Asvq24* for *Parabacteroides* (Bacteroidetes), Asvq25 for *Lactobacillus* (Firmicutes), Asvq9 and Asvq10 for the *Streptococcus* genus (Firmicutes), Asvq26 for the family of *Clostridiales vadinBB60* (Firmicutes), and Asvq27 for the *Lachnospiraceae* family (Firmicutes). This provides further support for previous findings suggesting that the ratio of Firmicutes to Bacteroidetes is relevant to metabolic disease states. Diet is the strongest predictor for these ASV except for the abundances of *Parabacteroides*, *Lactobacillus*, and *Clostridiales vadin BB60* (all Firmicutes)*.* The gene-by-diet interaction is most prominent for these three exceptions. Diet-specific QTL are the most clinically actionable observations as they identify a subgroup of the population that would be sensitive to a dietary intervention to modify the microbial trait. Loci identified by the sex- and diet-independent QTL and QTL by sex models are informative but do not provide the same type of direct avenue for intervention.

The American diet contains multiple sources of animal proteins, some of which contribute to the total fat content of the diet, while the main protein source in the ketogenic diet is casein. The fat component of the ketogenic diet is composed of equal parts butter and lard with a small portion of corn and menhaden oils, while the fat component of the American diet is a more diverse mixture of primarily butter as well as corn, menhaden, flaxseed, olive oil, and fat derived from the animal proteins. Lard-derived fat has been shown to reduce the abundance of *Streptococcus* [11]. This may explain, in part, why we observe that FVB alleles on the American diet are associated with higher abundances of two *Streptococci* ASV.

For the only loci picked up by the QTL by sex model, *Asvq28* for *Alistipes*, the strongest predictor in the model was again diet. Sex specificity for abundance of *Alistipes* has been established in studies of pre- and post-menopausal women and men. Men were more likely than pre- or post-menopausal women to have higher abundances of *Alistipes* in their fecal samples [42]. The realized importance of sex as a biological variable has increased attention paid to the role of steroid hormones in development of obesity and Metabolic Syndrome [23]. Plasma testosterone has also been linked to microbial features in men, and the post-menopausal microbiome becomes more similar to the male microbiome over time [42].

QTL for *Rikenella* (*Asvq7*), *Ruminiclostridium* (*Asvq16*), *Bilophila* (*Asvq17*), Bray–Curtis PCo2 (*Bcpc2q*), fat mass gained during the feeding trial (*Fmgq1*), and serum HDL cholesterol concentration (*Hdlq1*) overlap on the distal part of Chr 1 and QTL for the *Rikenelleceae RC9* Gut Group (*Asvq23*), *Parabacteroides* (*Asvq24*) and weighted UniFraction PCo1 (*Wufpc1q*) overlap on Chr 16. American or westernized diets are associated with increased abundances of *Bilophila wadsworthia*, which coincides with increased LDL cholesterol concentration and links this species of *Bilophila* to dyslipidemia and increased inflammation [43]. Gut microbiota signatures from overweight and obese patients have been associated with significant decreases in *Rikenella* and *Parabacteroides* species as well as increases in *Ruminococcus* species in the same subjects [44]. *Rikenellaceae RC9 gut group* has been associated with lipid metabolism in response to high fat diets [15, 45]. Previous associations between these organisms and metabolic traits make the overlapping loci associated with them higher priority for future analyses.

As mentioned previously, gut microbiota utilizes nutrients passing through the gastrointestinal tract. Microbial metabolism of these nutrients produces metabolites and microbial-derived metabolites known to impact metabolic health [1]. These metabolites may represent latent variables linking the genomic region underlying *Fmgq1*, *Hdlq1*, *Bcpc2q*, *Asvq7*,* Asvq16*, and *Asvq17* and each of their associated traits as well as *Wufpc1q*, *Asvq23*, and *Asvq24* and their associated traits.

The SEM for traits mapping to distal Chr 1 illustrated direct effects of the FVB/FVB genotype at *Bcpc2q* increasing abundance of *Bilophila* and *Ruminiclostridium 9*. A covariance pathway was detected between *Bcpc2q* and *Rikenella* and while the directionality of this relationship was not defined by the model, this suggests that the FVB/FVB genotype at *Bcpc2q* also increased the abundance of *Rikenella*. We observed direct effects of *Bilophila*, *Ruminiclostridium 9* and *Rikenella* on Bray Curtis PC2 in addition to the direct effect of diet. In addition to the direct effects of *Bilophila* and *Ruminiclostridium 9*, we identified a covariance pathway between these organisms that likely contributes to the overall relationship of these microbiota with Bray–Curtis PC2. While the microbiota and metabolic traits appear to be independently linked to *Bcpc2q*, another covariance pathway is observed between Bray–Curtis PCo2 and the amount of fat mass gained during the feeding trial. Taken together, these observations suggest that *Bilophila*, *Ruminiclostridium 9*, and *Rikenella* are driving differences in microbial beta diversity represented in Bray–Curtis PCo2, and the overall composition of the microbiome may be correlated with the amount of fat mass gained during the feeding trial. Species that other species in an ecosystem rely heavily upon are referred to as keystone species and drive diversity within the ecosystem [46, 47]. These results suggest that *Bilophila*, *Ruminiclostridium 9*, and *Rikenella* are candidate keystone species. The covariance pathway observed between the Bray–Curtis index PCo2 and the amount of fat mass gained might reflect a more complex relationship between the overall composition of the gut microbiota and its effects on metabolic features.

The SEM for traits mapping to distal Chr 16 illustrated direct effects of *Wufpc1q* interacting with diet on the abundances of *Rikenellaceae RC9 Gut Group* and *Parabacteroides* as well as a direct effect of diet and *Parabacteroides* on weighted UniFraction PCo1. Parabacteroides is a diet-specific, candidate keystone species. The observed a covariance pathway between these two organisms suggests *Rikenellaceae RC9 Gut Group* may be an additional candidate keystone species driving differences in the composition of the microbiota in a diet-specific manner.

Fluctuations in the abundance of these organisms would have dramatic consequences on other organisms in the ecosystem. *Bilophila*,* Ruminicostridium 9* and *Rikenella* represent candidates for keystone species among the organisms mapping to the distal region of Chr 1. Their direct effects on Bray–Curtis PCo2 detected in the structural equation model suggest abundances of these organisms drive differences in beta diversity. The proposed model lends itself to this speculation if the abundance of *Bilophila* has consequences for bile acid composition and abundances of other microbiota in the large intestine as described by others [48, 49]. We have previously demonstrated that the FVB/FVB genotype drives higher serum HDL cholesterol concentration at the locus *Hdlq1*, likely through Apolipoprotein A2 (*Apoa2*) [23]. *Apoa2* is also a primary candidate gene of interest within the confidence intervals for *Asvq7*, *Asvq16*, *Asvq17*, and *Bcpc2q*. HDL cholesterol is a preferred precursor to bile acid synthesis and secretion [50]. Despite there being no direct relationship observed between abundance of these organisms and metabolic traits in the current model, these basic biological associations leave ample space for future analyses into what is likely a more complex network of latent variables tying together these microbial and metabolic traits. Additional candidate genes of interest that are expressed in the intestines were identified within the *Bcpc2q* interval (*F11r*, *Fcer1g*, *Ndufs2* and *Sdhc)*. *F11r* and *Fcer1g* are found on KEGG pathways primarily related to the immune system while *Ndufs2* and *Sdhc* are found on the Non-alcoholic fatty liver disease pathway (mmu04932).

*Parabacteroides* was also identified as a candidate keystone species among the organisms mapping to the distal part of Chr 16. We were unable to narrow the list of positional candidate genes at *Wufpc1q* harboring non-synonymous transcript variants with the KEGG pathways included in the query. However, the vast majority of genes at *Wufpc1q* were annotated with the epithelial barrier and immune system pathways such as Tight junction (mmu04530) and Inflammatory bowel disease (mmu05321) and related pathways. Other types of variants were present in genes within the Wufpc1q confidence interval such as, synonymous transcript variants and intronic variants which may be of interest in future analyses. For example, *Sod1* harbors an intron variant that diverges between the two strains and has previously been associated with both the ratio of Firmicutes to Bacteroidetes as well as obesity, providing direct evidence for variants in *Sod1* regulating microbial diversity and a possible link to metabolic traits like obesity [51, 52]. Of note, *Parabacteroides* and *Rikenelleceae RC9 gut group* both belong to the Bacteroidetes phylum. Future work will focus on confirming causal relationships between candidate keystone species and measures of beta diversity.

Our report is limited in part by the choice to generate a unidirectional F1 and subsequently, F2 population. This precludes the ability to identify epistatic interactions between the autosomal genome and either the Y chromosome or the mitochondrial genome because only the B6 mitochondrial genome and FVB Y chromosome is present in our F2 population. However, this makes us more certain that the findings we have reported are not driven by the paternal chromosome nor the mitochondria. Also, the choice of the 16S V4 region per the EMP 16S analysis protocol described in the methods may limit which bacteria are identified. However, we were careful to report taxonomic assignments only up to the genus level with the understanding that taxonomic assignments after the genus level using a small 16S V4 region are not always reliable.

## Conclusions

The current experiment identified organisms for which irrespective to genetic background, diet was the strongest predictor of gut microbiota, organisms for which combinations of sex, diet, and genotypes predictor the gut microbiota, as well as a single organism for which genetic background was the strongest predictor for bacterial, *Coriobacteriaceae UCG-002*. These results demonstrate the effect that sex, diet, and genetic background have on inter-individual differences in gut microbiota. While diet and genotype-dependent QTLs for microbial abundance are the most clinically relevant regarding efforts to advance precision nutrition, diet-dependent observations are likely related to specific ingredients in the diets which makes these observations heavily context dependent and difficult to recapitulate from investigator to investigator when non-human comparable ingredients are used in the preclinical setting. We observed that nearly all microbial QTL, even those that were identified under the QTL and QTL by sex models, were potently influenced by diet. As such, care should be taken to utilize diets composed of diverse ingredients in preclinical trials to better recapitulate the host-microbiome environment in humans. Precision nutrition will be advanced through integration of genetic variation, microbiota variation, and sex in response to diets varied in carbohydrate composition to elucidate the composition of the “ideal” microbiome and personalized interventions to achieve that composition.

### Supplementary Information

**Additional file 1: Supplementary Figure S1.** Genetic Map. 1,667 markers that were polymorphic between B6 and FVB were used for the association analyses. **Supplementary Figure S2.** Jaccard Index. PC1 and PC2 describe 21.3% and 10.6% of the variation in ASV respectively. **Table S1.** Diet compositions. **Table S2.** ARRIVE criteria used in the study. **Table S3.** Comprehensive list of all KEGG querie. **Table S4.** Effect of sex and diet on phyla abundance.

## Data Availability

16S V4 Sequences are publicly available on the SRA database under the Bioproject ID “PRJNA803237.”
