# Supplementary material for: Analysis of strain, sex, and diet-dependent modulation of gut microbiota reveals candidate keystone organisms driving microbial diversity in response to American and ketogenic diets
Source: Microbiome. 2023 Oct 3;11:220. doi: 10.1186/s40168-023-01588-w (PMC10546677; doi:10.1186/s40168-023-01588-w)
Supplement: Supplementary file 2 — Additional file 1: Supplementary Figure S1. Genetic Map. 1,667 markers that were polymorphic between B6 and FVB were used for the association analyses. Supplementary Figure S2. Jaccard Index. PC1 and PC2 describe 21.3% and 10.6% of the variation in ASV respectively. Table S1. Diet compositions. Table S2. ARRIVE criteria used in the study. Table S3. Comprehensive list of all KEGG querie. Table S4. Effect of sex and diet on phyla abundance. [file 40168_2023_1588_MOESM1_ESM.pdf]

Supplementary Figures

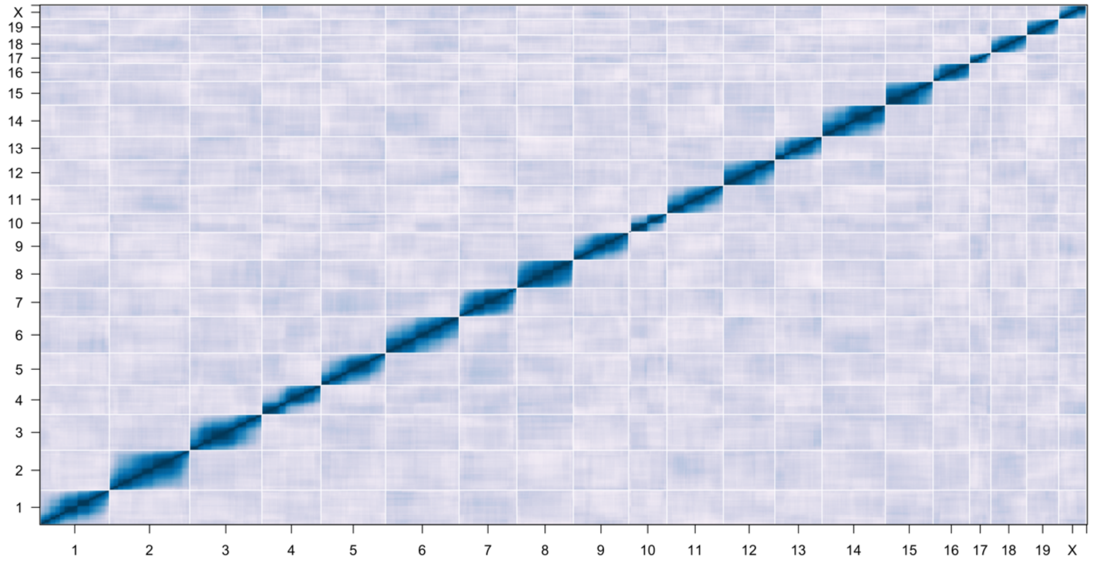

Figure S1.

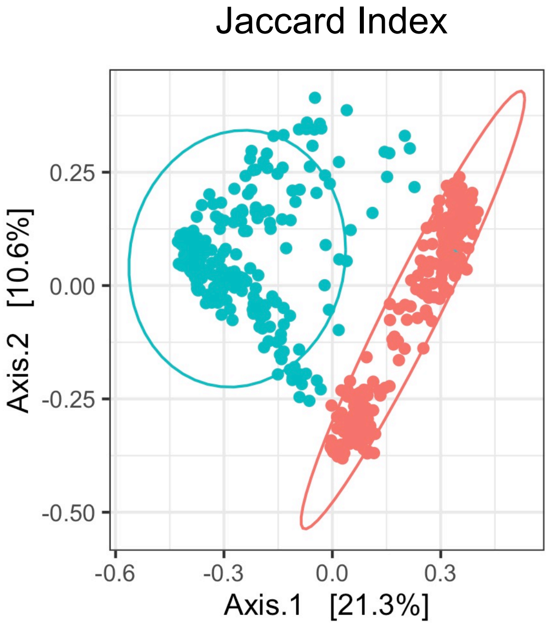

Figure S2.

Table S1. Diet compositions.

	D12052705C (American)	D12052706 (Ketogenic)
Ingredient	(g)	(g)
Casein	44.5	262.5
Soy Protein Isolate, Supro 661	0	0
Fish Protein Isolate	9.8	0
Egg White	63.6	0
Beef, Cooked	88.9	0
L-Cystine	3.5	4.9
Corn Starch	34.7	0
Wheat Starch	225.5	0
Potato Starch	34.7	0
Sucrose	237.1	0
Fructose	25.4	0
	0	0
Cellulose, BW200	21.0	61.5
Inulin	6.9	20.5
Corn Oil	39.8	13.1
Menhaden Oil (299 ppm tBHQ)	1.2	13.1
Butter, Anhydrous	62.6	264.1
Lard	0	264.1
Flaxseed Oil	1.2	0
Olive Oil	31.8	0
t-BHQ	0	0
Mineral Mix S10026	11.6	16.4
Dicalcium Phosphate	15.0	21.3
Calcium Carbonate	6.4	9.0
Potassium Citrate, 1 H2O	19.1	27.1
Vitamin Mix V10001	11.6	16.4
Biotin (1%)	0.1	0
Choline Bitartrate	2.3	3.3
Cholesterol	1.7	2.5

FD&C Red Dye #5	0	0
FD&C Blue Dye #1	0	0
Total	1000	1000
(g)		
Protein	162.7	233.3
Carbohydrate	526.5	5.1
Fat	161.5	557.6
Cholesterol	2.1	3.4
Fiber	26.3	76.9
% of Total Weight (g)		
Protein	16.3	23.3
Carbohydrate	52.6	0.5
Fat	16.2	55.8
Cholesterol	0.21	0.3
Fiber	2.6	7.7
Energy (kcal)		
Protein	651.1	933.5
Carbohydrate	2105.8	21.3
Fat	1453.6	5018.4
Total	4210.4	5973.2
% of Total Energy (kcal)		
Protein	15	16
Carbohydrate	50	0
Fat	35	84
% of total weight (g)		
Contribution to Protein (g)		
Casein	23.8	97.9
Fish Protein Isolate	5.7	0
Egg White	31.7	0
Beef, Cooked	36.6	0
L-Cystine	2.1	2.1
% of total weight (g)		
Contribution to Carbohydrate		
Corn Starch	5.9	0
Wheat Starch	38.4	0

Potato Starch	5.3	0
Sucrose	45.6	0
Fructose	4.9	0
Lipid Composition		
C2, Acetic	0	0
C4, Butyric	2.0	8.5
C6, Caproic	1.2	5.1
C8, Caprylic	0.7	3.0
C10, Capric	1.6	6.7
C12, Lauric	1.7	7.7
C14, Myristic	7.1	30.4
C14:1, Myristoleic	1.2	3.9
C15:0	0.1	0.3
C16, Palmitic	30.0	123.5
C16:1, Palmitoleic	2.8	11.0
C16:2	0	0.2
C16:3	0	0.2
C16:4	0	0.2
C17:0	0.2	1.0
C17:1, n-9	0.2	0
C18, Stearic	11.8	60.5
C18:1, Oleic, n-9	58.4	157.7
C18:1, Vaccenic, n-7	1.0	0
C18:2, Linoleic, n-6	30.6	78.6
C18:3, gamma-Linolenic, n-6	0	0
C18:3, alpha-Linolenic, n-3	2.3	7.5
C18:4, Stearidonic, n-3	0	0.3
C20, Arachidic	0.8	3.0
C20:1, n-9	0.2	1.8
C20:2, Eicosadienoic, n-6	0	2.1
C20:3, n-6	0	0.3
C20:3, n-3	0	0
C20:4, Arachidonic, n-6	0.1	1.0
C20:4, n-3	0	0
C20:5, Eicosapentaenoic, n-3	0.1	1.8
C21:0	0	0
C21:5, n-3	0	0.2
C22, Behenic	0	0
C22:1, Erucic	0	0

C22:4, Clupanodonic, n-6	0	0
C22:5, n-3	0	0.7
C22:5, n-6	0	0
C22:6, Docosahexaenoic, n-3	0.1	1.3
C24, Lignoceric	0	0
C24:1	0	0
Lipid Profile		
Saturated (g)	56.8	248.2
Monounsaturated (g)	62.6	174.6
Polyunsaturated (g)	33.5	94.0
Saturated (%)	42.4	78.6
Monounsaturated (%)	46.8	55.1
Polyunsaturated (%)	25.1	29.7
Total Omega-6 (g)	30.9	82.2
Total Omega-6 (%)	23.1	26.1
Total Omega-3 (g)	2.8	11.8
Total Omega-3 (%)	2.1	3.8
n6 : n3 ratio	13.1	11.3

Table S2. ARRIVE criteria used in the study.

ARRIVE Husbandry Guidelines	Details
Cage	NexGen Mouse 500 (NexGen Individually Ventilated Cage System); 77.5 in^2 / 500 cm^2 for mouse housing, Low Vibration.
Food	The American diet is a powdered meal composed of 35% of energy from fat, 50% from carbohydrate and the ketogenic diet is a paste composed of 84% of energy from fat, 0% from carbohydrates). Neither diet was irradiated. See Supplementary Table S1 for additional details and product information.
Water	Reverse osmosis water; not acidified or supplemented. Water bottles are changed weekly with cage changes and when they get below 150 mL.
Bedding	Sani-Chips (Lab Supply). The chips are dried to 8% (+/-2%) moisture content and then screened to National Institute of Health specifications, which include a size range from 8 to 20 mesh.
Nesting Material	Ancare Item# NES3600 (Lab Supply). Neslets are sterilized pulped virgin cotton fiber which is chemically inert, odorless and non-ingestible.
Temperature and Humidity	22 °C; Humidity is not monitored/regulated.
Sanitation	Cage change once per week. Sentinels are tested quarterly. A Clinical Serology panel is used in quarters 1-3, and a Basic Serology panel is used in quarter 4. Every quarter, mice get a complete necropsy, with fur swabs and fecals sent off to IDEXX for PCR testing of pinworms and fur mites. Random cecal exams are performed each quarter on about 25-30% of the sentinels tested.<br>**Clinical Serology:** Mycoplasma pulmonis, EDIM, MHV, MNV, MPV, MVM, Sendai, and TMEV<br>**Basic Serology:** Everything in the clinical serology plus Ectromelia, LCMV, PVM, and REO3
Social environment	Up to 5 mice per cage
Biosecurity (level)	BSL1
Lighting	12-hour light cycle
Environmental Enrichment	Wood Gnawing Blocks, Certified (Bio-Serv, Product K3511-300).
Sex of the Experimenter	Both male and female experimenters and caretakers.

Table S3. Comprehensive list of all KEGG querie

Inclusion Criteria	KEGG id
Glucose and insulin related pathways	mmu00010
	mmu04910
	mmu04931
Diabetes Mellitus related pathways	mmu04940
	mmu04930
	mmu04950
Fatty acid and adipocyte related pathways	mmu00061
	mmu04920
	mmu04923
Digestion and absorption related pathways	mmu04973
	mmu04974
	mmu04975
Cholesterol related pathways	mmu04979
	mmu00120
	mmu04976
	mmu03320
	mmu04152
	mmu04927
	mmu00140
	mmu04913
	mmu00100
	mmu04925
	mmu04934
Additional Obesity and Metabolic Syndrome related pathways	mmu04146
	mmu04932
	mmu04714
	mmu04371

Epithelial Barrier and related pathways	mmu04530
	mmu04066
	mmu05143
Immune system and related pathways	mmu05320
	mmu04672
	mmu05235
	mmu05322
	mmu05162
	mmu04622
	mmu05150
	mmu04613
	mmu04657
	mmu04514
	mmu05163
	mmu03050
	mmu04062
	mmu04620
	mmu04650
	mmu04659
	mmu05152
	mmu05323
	mmu05340
	mmu05146
	mmu05171
	mmu05140
	mmu05164
	mmu05168
	mmu05416
	mmu04061
	mmu04380
	mmu04621
	mmu04623
	mmu04658
	mmu04660
	mmu04670
	mmu04940
	mmu04978
	mmu05160
	mmu05167
	mmu05321

	mmu05330
	mmu05414
	mmu05132
	mmu05165
	mmu05170
	mmu04612
	mmu04917
	mmu05133
	mmu05135
	mmu05161
	mmu05169
	mmu05203

Table S4. Effect of sex and diet on phyla abundance.

Phylum	Sex (p-value)	Diet (p-value)	Sex*Diet (p-value)	% Variance Explained by Sex	% Variance Explained by Diet	% Variance Explained by Sex*Diet
Actinobacteria	0.246	< 0.001	0.209	0.11	64.79	0.13
Bacteroidetes	0.315	< 0.001	0.794	0.18	25.49	0.01
Cyanobacteria	0.055	< 0.001	0.137	0.82	3.56	0.5
Firmicutes	0.411	< 0.001	0.330	0.06	61.22	0.09
Proteobacteria	0.814	< 0.001	0.638	0.01	3.02	0.05
Verrucomicrobia	0.031	< 0.001	0.708	0.99	9.21	0.03
